# Herpes simplex virus type 2 encephalitis in a child with chronic progressive white matter lesions: A case report

**DOI:** 10.1097/MD.0000000000032289

**Published:** 2022-12-16

**Authors:** Lei Xie, Aijun Lu, Xiaoling Wang, Bihong Cheng, Xuemin Zhu, Huaiqiang Hu

**Affiliations:** a School of Clinical Medicine, Weifang Medical University, Weifang, China; b The 960th Hospital of PLA, Jinan, China.

**Keywords:** brain biopsy, encephalitis, granulomatous, Herpes simplex virus type 2, magnetic resonance

## Abstract

This case is a rare manifestation of central nervous system infection of Herpes simplex virus (HSV)-2. Due to few studies in China, it provides a pathological basis for further diagnosis and treatment of HSV-2.

**Patient concerns:**

We describe a patient with HSV-2 virus infection who was diagnosed with HSV-2 encephalitis in a Chinese patient.

**Diagnosis:**

Based on brain biopsy and pathological findings, the patient was diagnosed with HSV-2 encephalitis.

**Interventions:**

Hormone and antiviral therapy were given.

**Outcome:**

The patient eventually died.

**Lessons:**

The diagnosis and differential diagnosis of the disease is very difficult. Its differential diagnosis include cerebrovascular disease, bacteria or fungi and other viral infection of the brain.

## 1. Introduction

Herpes simplex encephalitis (HSE) is the most common sporadic encephalitis worldwide, accounting for about 10 to 20% of all viral encephalitis.^[1]^ Although Kupila et al^[2]^ showed that herpes simplex virus (HSV)-2 was the second most common cause of viral meningitis, as confirmed through microbial findings, HSV-2 is relatively rare in encephalitis compared with HSV-1, only 10% of cases are caused by HSV-2^[3]^ and most of these occur in neonates.^[4]^ The lesion site mainly involves the brain stem,^[5]^ with little impact on the temporal or orbitofrontal lobe. Its symptoms include altered consciousness level, cranial nerve damage, hemiplegia, and partial sensory loss,^[6]^ the clinical manifestations are nonspecific, and diagnosis may be difficult. HSV-1 encephalitis usually shows progressive deterioration, while HSV-2 encephalitis mostly has a benign course.^[7,8]^ cranial magnetic resonance examination may be normal or show nonspecific white matter lesions. Acyclovir is common effective on HSV; delays in diagnosis and treatment will greatly increase the transmission of infection along the axons, leading to complex conditions.^[9]^ In this report, a case of HSV-2 encephalitis was analyzed in a child with progressive stroke-like manifestations. A magnetic resonance imaging (MRI) of the head showed progressive white matter lesions. Acyclovir had poor therapeutic effects. The pathological mechanism was further explored through brain biopsy.

## 2. Background

**Figure 1.**
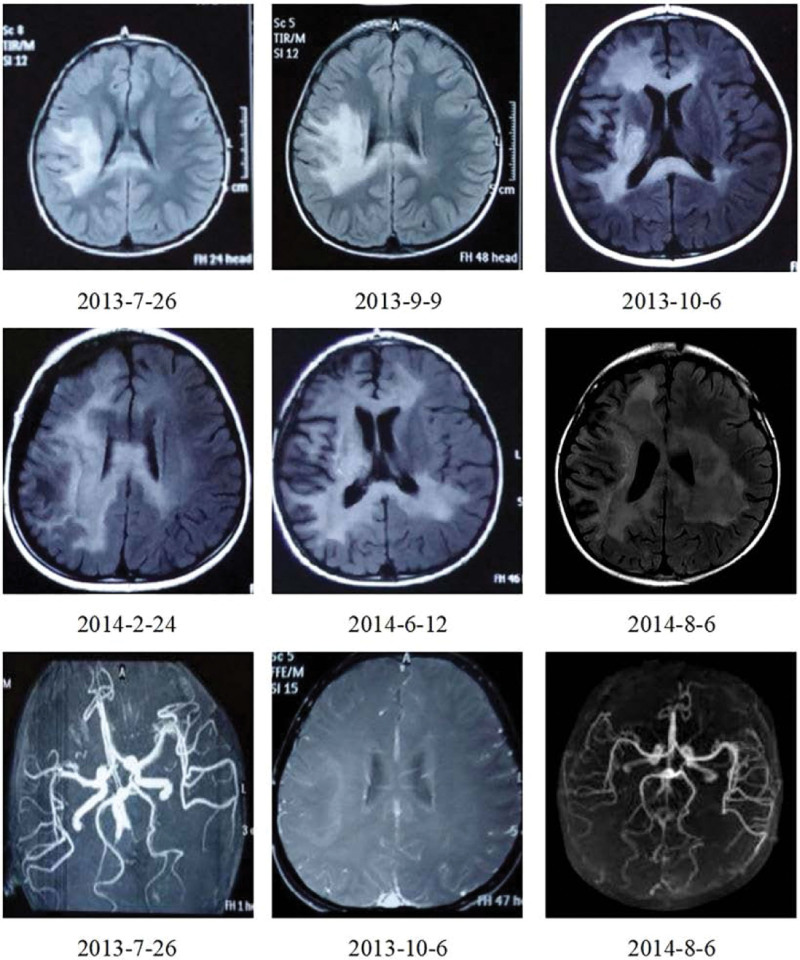
Brain magnetic resonance imaging and angiography: Multiple abnormal signals, enhancement foci in brain parenchyma and meninges, and progression.

**Figure 2.**
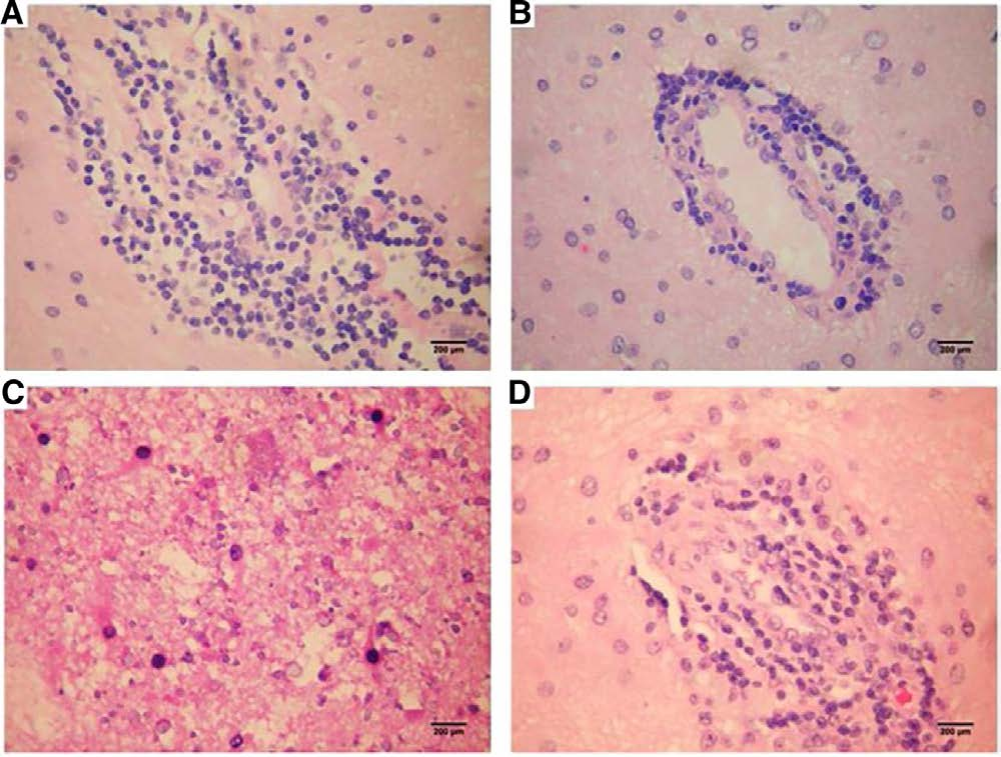
Haematoxylin and eosin staining (A–B, ×400): no pia mater hypertrophy, mild vascular dilatation, and no inflammatory cell infiltration in the meninges and perivascular cortex. Degeneration and loss of neurons, mild proliferation of glial cells, a small amount of microhaemorrhage, and cuff-like changes in the mononuclear cells, mainly in the lymphocytes, can be seen around the venules. The white matter showed highly loose changes, and cavitation formation was observed. Klüver-Barrera staining (C–D, ×400): obvious demyelination of the myelin sheath, granulomatous changes formed by the infiltration of mononuclear cells around the small vessels in many spots, no typical multinucleated giant cells, and no nuclear abnormality or inclusion body formation were observed. Glial cells and infiltrating inflammatory cells of the surviving neurons were normal in morphology.

**Figure 3.**
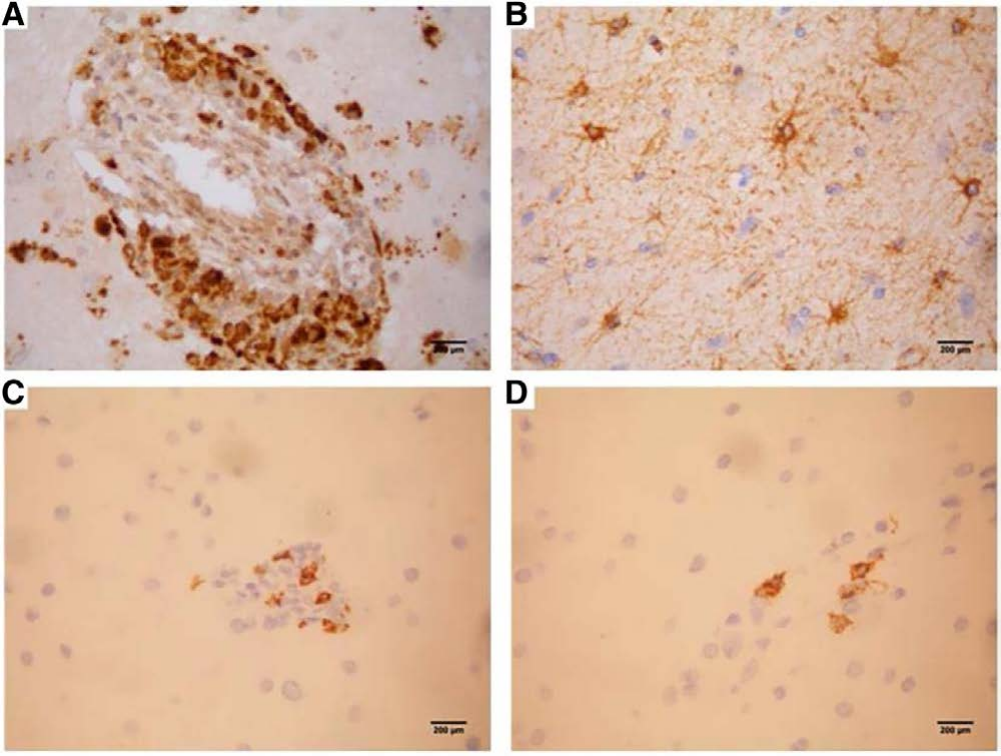
Immunohistochemical dyeing (×400): positive for (A) CD68 and (B) glial fibrillary acidic protein, and weak positive for (C) CD3 and (D) CD20.

**Figure 4.**
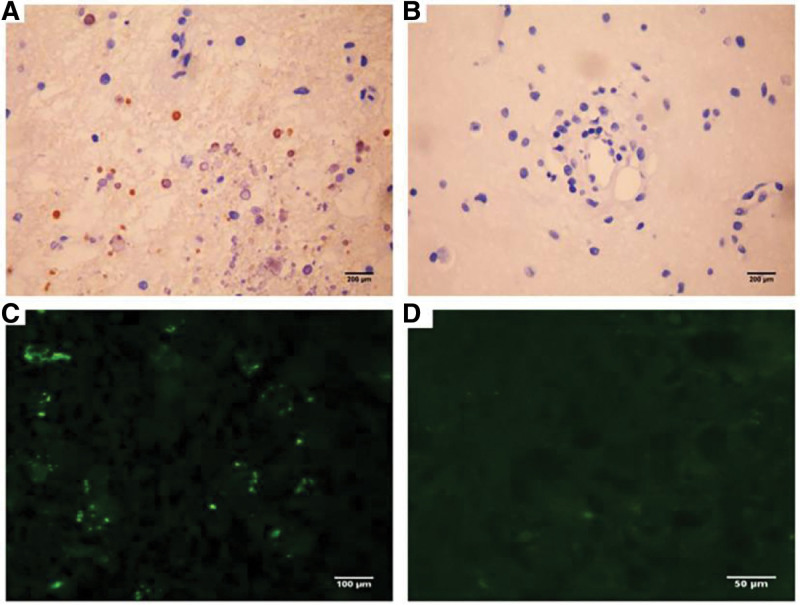
Immunohistochemical dyeing (×400): (A) HSV-2-positive, (B) HSV-1-negative; Fluorescence in situ hybridization (×400): (C) HSV-2-positive, (D) HSV-1-negative. HSV = herpes simplex virus.

The patient was a 5-years-old boy with normal immune capacity. He gradually experienced left limb weakness after catching a cold and fever, with his hands usually balled into fists. Brain MRI showed multiple abnormal signals in the white matter; magnetic resonance angiography was normal (Fig. 1). Serum autoimmune antibody repertoire, streptococcal and tuberculosis-related antibodies, and tumor markers were negative. blood organic acid metabolism and mitochondrial encephalopathy genetic screening were normal. The HSV IgM antibodies have an index value of 1.56 (> 1.1 is positive), while IgG have an index value of 10.1 (> 1.1 is positive). A lumbar puncture indicated that the cerebrospinal fluid (CSF) was red and transparent, the number of white blood cells in the CSF was 27 × 10^6^/L, CSF IgM was 0.472 mg/dL (0–0.13 mg/ dL), and CSF myelin basic protein was elevated. Anti-virus antibody, pathogenic microbial DNA, anti-N-methyl-D-aspartate receptor antibody in serum and cerebrospinal fluid,and oligoclonal bands were negative. The electromyogram and auditory evoked potential test results were normal; the electroencephalogram results showed mild abnormalities, and the visual evoked potential showed slightly prolonged bilateral P100 latency. we considered HSV infection, demyelinating encephalopathy. The patient was treated with acyclovir, intravenous immunoglobulin and methylprednisolone sodium succinate. The effect was poor, and the symptoms progressively worsened. Furthermore, multiple abnormal signals appeared, and abnormal enhancement lesionwere observed in the brain parenchyma and meninges, and the lesions were larger than before with developing symptoms (Fig. 1). Multiple intracranial abnormal signals appeared on the 3^rd^ month after onset (Fig. 1). A brain biopsy was performed 6 months later, and pathology results showed inflammatory lesions in the brain tissue. The specimens were re-stained, and staining with hematoxylin and eosin showed the degeneration of neurons, loss of gangophil, and formation of cuff-like lymphocytes around the venules. Klüver-Barrera (KB) staining showed the obvious demyelination of the myelin sheath, and many granulomatous mononuclear cells infiltrated around the small vessels. Both immunohistochemical staining and fluorescence in situ hybridization showed HSV-2-positive and HSV-1-negative results (Figs. 2–4). After discharge, the patient still had difficulty eating, and his symptoms worsened. Consequently, the patient eventually died.

## 3. Discussion

HSV is neurotropic and latent in the trigeminal ganglion.^[10]^ After reactivation, it spreads through the axons and infects the brain and blood vessels, resulting in axon and glial damage because the combination of viral replicatolytic cell and immune-mediated mechanism.^[11]^ In relation to HSV-1 encephalitis, HSV-2 is rare, causing only 10% of cases.^[3]^ the underlying pathology is a necrotizing inflammatory process, which may be caused by cytokines, chemokines, and proteases, causing the weakening of neurovascular units.^[12–14]^ HSV causes a wide range of central nervous system diseases including brain stem encephalitis, meningitis, myelitis, recurrent meningitis, consciousness disorder, cranial nerve damage, hemiplegia and partial loss of feeling in the body. early diagnosis is difficult now, and based on previous research, mortality still ranges from 7% to 17% even with appropriate acyclovir treatment.

The CSF, etiological analysis, and cranial MRI are helpful in the diagnosis of HSE. The white blood cell count and protein are normal or slightly increase in CSF; The absence of these features does not preclude a diagnosis of HSE. HSV IgM and IgG were both positive without specific classification 1 week after onset in this case, and the number of CSF cells was significantly increased; pathogenic microorganism DNA was negative. Immunohistochemistry and fluorescence in situ hybridization of the brain biopsy specimens showed positiveHSV-2, negative HSV-1.The microbiological DNA screening in the early course was negative, which may be related to the low viral replication load. Regardless, the positive HSV IgM of CSF indicates that there is indeed an acute infection.

Cranial MRI is considered to be a more sensitive method to detect HSV lesions. it usually shows significantly abnormal results even after normal CT examination.^[15]^ A variety of abnormalities may be shown including localized edema and punctate or massive bleeding, usually in the cortex or shallow white matter.^[16]^ The patient in this report had certain imaging particularities such as the progressive white matter lesions similar to acute disseminated encephalomyelitis, which were mainly concentrated in the bilateral frontotemporal lobes and brain stem. For chronic progressive neuroimaging changes, immune-mediated tissue damage is currently believed to be the main mechanism.^[17]^ although the imaging changes progressed, this patient had no new clinical symptoms and only presents with aggravated existing stroke-like symptoms; abnormal progressive signals may sometimes not be consistent with the activity or persistence of the disease. By all means, longitudinal MRI studies in children with asymptomatic HSE have shown self-limited diffuse white matter lesions.^[18]^ Their white matter lesions and 4 cases of HSE with similar white matter lesions were compared, and the results showed that the pathological mechanism can be divided into the: subacute delayed progress of the damage to the focal myelin sheath surrounding the blood vessels,^[19]^ which is widely associated with gray matter without the acute phase of pathological changes; and a kind of pathological change in treatment without symptoms or disappear automatically.^[20]^ In this case, the level of CSF myelin basic protein increased, KB staining showed myelin depigmentation, supporting myelin destruction. white matter showed highly loose changes, and cavity formation was visible. Thus, we speculated that the white matter lesions of head MRI was the pathologic mechanism for the former; the pathological results include small vascular inflammation and granuloma formation. diffusion-weighted imaging showed high signals, and histology results indicated cytotoxic edema, accompanied by micro hemorrhage and necrosis.^[21,22]^ in addition to the direct invasion necrosis caused by viral encephalitis, Studies showed that HSV-2 presented with limited diffusion and watershed cerebral infarctions in areas far from the infected parts of the brain, thus increasing the likelihood of low perfusion damage.^[23,24]^ Therefore, approximately 50% of patients experienced disease progression despite acyclovir therapy. The association between the occurrence of stroke-like lesions and varicella-zoster virus (VZV) infection has been reported.^[25]^ VZV can cause vascular endothelial injury and thrombosis, promote the subcutaneous proliferation of smooth muscle cells, fibroblasts and collagen,^[26]^ and present with microbleeding changes. Thus, further studies should be performed on whether the same or similar mechanisms mentioned above also present in HSV-2. stroke caused by adult HSV-2 encephalitis,^[27,28]^ the lesion changes are secondary to the endothelial injury caused by virus-induced small vasculitis.^[29]^ A study^[30]^ performed a brain biopsy on an asymptomatic child, wherein brain MRI T2-weighted images showed granulomatous white matter inflammation with multinucleated giant cells, focal vasculitis, necrosis, microcalcification, and hemosiderin deposition, as well as abnormal temporal white matter in fluid attenuated inversion recovery. After acyclovir antiviral treatment, polymerase chain reaction (PCR) detection of virus was negative; progressive imaging procedures were still conducted. Glucocorticoid therapy had an effect on the lesions, suggesting that the white matter lesions may be vasculoinflammatory lesions after a viral infection.

The mechanism of HSV-2 and intracranial vascular inflammation is not yet clear. As previously mentioned, VZV infection of the central nervous system can lead to vascular lesions.^[26]^ When the latent HSV in the ganglion is reactivated, it spreads to the adventitia through the axon, blood vessel walls, and eventually damages the adventitia,^[31]^ resulting in the fibrinoid necrosis of the blood vessel walls, intimal hyperplasia, loss of elastic plate, and inflammatory infiltration of peripheral lymphocytes or monocyte aggregation. Thus, such vascular destruction may be secondary to a direct viral invasion or some immune response associated with the invasion. The mechanisms of immune reaction-related injury include the production of membrane attack complex and associated inflammation caused by antibodies directly attacking the immune complex. However, studies on the correlation between HSV-2 and vascular lesions is currently lacking.

Brain biopsy showed the infiltration of perivascular lymphocytes and macrophages, necrotizing granulomatous inflammation, multinucleated giant cells and calcification in HSE, which is rarely seen in patients with progressive development which mainly presents with mononuclear cell infiltration and granuloma formation without multinucleated giant cells. Thus, mechanisms may be unclear and may be related to a particular inflammatory pathway of HSV-2 infection. Pathological observations include veinlet changes in peripheral lymphocytes, several small perivascular mononuclear cells infiltration forming granulomas, no nuclear abnormalities, and inclusion body formation, which are a phenomenon of active and ongoing disease processes. Pathologic descriptions of progressive HSE are rare, and the features of the granuloma are uncommon. Combined with the results of immunohistochemical staining, which suggests an active vascular inflammatory process, and immune-mediated tissue damage may be involved in the lesion formation. This patient had a long course of disease, and repeating hormone and antiviral therapy were ineffective. Moreover, the therapeutic effect was still not obvious, and the disease further progressed. It was speculated that multiple hormonal therapy may aggravate the initial direct viral invasion and damage. Subsequently, immune-mediated inflammation played a more important role or was superposed with the direct viral damage, eventually forming lymphocyte cuff-like and granulomatous changes.

The diagnosis and differential diagnosis of the disease is very difficult. Its differential diagnosis include cerebrovascular disease, bacteria or fungi and other viral infection of the brain, acute disseminated encephalomyelitis,^[15]^ mitochondria encephalopathy, human herpesvirus 6 infection, deputy tumor edge encephalitis, listeria disease, and voltage-gated potassium channel antibody disorders, which has similar lesions as HSE with pathological changes. Thus, there is a need to better identification; however, most can be distinguished by a head MRI and brain biopsy. Now the next generation sequencing of the genes of the pathogenic microorganisms in cerebrospinal fluid is of great significance for the identification of infections.

HSV-2 encephalitis with chronic progressive white matter lesions, especially in children, early clinical diagnosis is still difficult. MRI and CSF tests are helpful for its diagnosis, Current microbial gene sequencing is important for diagnosis, and biopsy is still important if the sequencing is negative or not consistent with the clinicand brain biopsy results can significantly contribute to understanding the disease’s mechanisms,diagnosis and treatment. immunohistochemical, in situ hybridization, and PCR assays can also help its diagnosis. There is currently no consensus regarding the duration and treatment of HSV-2 infection now. previous research has shown that HSV-2 infection is benign and self-limiting, understanding the clinical mechanism of HSV-2 encephalitis is crucial, especially in children wherein systematic studies are lacking, to recognize its symptoms and effects. This case presents the rare manifestations associated with HSV-2 central nervous system infection, as well as provides the pathological basis for its further diagnosis and treatment development since only a few studies have been reported in China.

## Acknowledgements

The authors gratefully acknowledge the patient and his family for allowing us to share their information.

## Author contributions

**Funding acquisition:** Xiaoling Wang, Xuemin Zhu.

**Project administration:** Bihong Cheng.

**Writing – original draft:** Lei Xie, Aijun Lu.

**Writing – review & editing:** Huaiqiang Hu.
